# Precise arraying of perovskite single crystals through droplet-assisted self-alignment

**DOI:** 10.1126/sciadv.ado0873

**Published:** 2024-07-10

**Authors:** Jianglei Zhang, Yifan Yang, Weijun Li, Zigao Tang, Zhiying Hu, Haotong Wei, Junhu Zhang, Bai Yang

**Affiliations:** ^1^ State Key Laboratory of Supramolecular Structure and Materials, College of Chemistry, Jilin University, Changchun 130012, PR China.; ^2^ Optical Functional Theranostics Joint Laboratory of Medicine and Chemistry, The First Hospital of Jilin University, Changchun 130012, PR China.

## Abstract

Patterned arrays of perovskite single crystals can avoid signal cross-talk in optoelectronic devices, while precise crystal distribution plays a crucial role in enhancing device performance and uniformity, optimizing photoelectric characteristics, and improving optical management. Here, we report a strategy of droplet-assisted self-alignment to precisely assemble the perovskite single-crystal arrays (PSCAs). High-quality single-crystal arrays of hybrid methylammonium lead bromide (MAPbBr_3_) and methylammonium lead chloride (MAPbCl_3_), and cesium lead bromide (CsPbBr_3_) can be precipitated under a formic acid vapor environment. The crystals floated within the suspended droplets undergo movement and rotation for precise alignment. The strategy allows us to deposit PSCAs with a pixel size range from 200 to 500 micrometers on diverse substrates, including indium tin oxide, glass, quartz, and poly(dimethylsiloxane), and the area can reach up to 10 centimeters by 10 centimeters. The PSCAs exhibit excellent photodetector performance with a large responsivity of 24 amperes per watt.

## Teaser

A strategy for self-alignment of perovskite crystals can achieve precise arrangement of single-crystal arrays.

## INTRODUCTION

Metal halide perovskites emerge as excellent candidates in diverse optoelectronic devices such as solar cells (*1*, *2*), photodetectors (*3*, *4*), radiation detectors (*5*–*7*), lasers (*8*, *9*), and light-emitting diodes (LEDs) (*10*, *11*). Although the efficiency of perovskite single-pixel device has steadily increased, the deployment of pixel arrays is necessary to adopt to modern technology in many cases such as large-area display, real-time imaging, and multipoint tracking (*12*–*16*). Compared with single-pixel devices, perovskite single-crystal arrays (PSCAs) exhibit advantages in large-area preparation, reducing signal cross-talk between pixels, high resolution, and flexible wearable device applications (*17*–*20*).

Traditional patterning methods include top-down and bottom-up processes (*21*). However, perovskite crystals are not compatible with common top-down patterning methods, such as lithography and electron beam lithography. The polar solvents used in lithography will dissolve or degrade perovskite crystals and the high-energy electron beam causes additional defects in crystal structure (*22*, *23*). Therefore, the construction of PSCAs basically follows the bottom-up principle, which includes template assembly, inkjet printing, and epitaxial growth. Precursor solution confinement through template assembly is a mainstream practice to array pixels by constructing periodic chemical modifications or physical topography on substrates (*24*–*35*), while inkjet printing can form a precursor droplet array directly on a uniform substrate (*20*, *36*–*38*). The driving force for growing PSCAs was in situ solvent evaporation or anti-solvent vapor diffusion. Vapor-phase epitaxy and solution-processed homoepitaxial growth can also be used to selectively grow PSCAs on pre-patterned substrates (*17*, *39*–*42*).

The regularity of crystal arrangement is a critical parameter for PSCAs, and achieving precise crystal alignment offers substantial advantages. This precision enhances device performance and uniformity by reducing structural defects (*43*–*45*), optimizes photoelectric properties (*46*, *47*), integrates seamlessly with existing technologies (*48*), and improves light management in photovoltaics (*49*), making it attractive for scalable and cost-effective production. During PSCA growth, the nucleation process plays a pivotal role in determining the final structure and properties of the crystals. However, the inherent randomness and uncertainty in nucleation make it challenging to precisely control crystal growth and arrangement. Researchers have devised innovative methods to enhance the accuracy of crystal arrangement. For instance, Lei *et al.* (*17*) reported a homoepitaxial growth method for preparing methylammonium lead bromide (MAPbBr_3_) single crystals with controlled locations, morphologies, and orientations. Peng *et al.* (*40*) successfully prepared single-crystalline perovskite films with controllable shapes, morphologies, and dimensions using a pattern-selective molecular epitaxial growth approach.

Here, we propose a droplet-assisted self-alignment approach for the precise assembly of perovskite single crystals, allowing for control over their morphologies, dimensions, positioning, and directional arrangement. This innovative strategy addresses the challenge of the regular arrangement of crystals arising from the inherent randomness and uncertainty of nucleation. Here, crystal nuclei undergo autonomous movement and rotation to achieve self-alignment following precipitation. The primary driving forces behind this alignment come from the crystal’s own gravitational force and the surface tension force of the precursor droplet. The benefits of this approach manifest in several key aspects: (i) Unlike previously reported methods, this strategy is uniquely suitable for the preparation of PSCAs containing crystals ranging from micrometers to submillimeters in size. This expanded range of crystal sizes opens up a broader range of potential applications for PSCAs, including ionizing radiation detection, which often requires thicker and more robust crystals. (ii) This strategy does not rely on the strict matching of crystals with a specific substrate. As a result, it allows for the preparation of diverse PSCAs on various substrates, such as indium tin oxide (ITO), glass, quartz, and poly(dimethylsiloxane) (PDMS). These characteristics provide greater flexibility and adaptability to different application scenarios, enabling more effective and efficient use of these arrays.

## RESULTS

### Droplet-assisted self-alignment of PSCAs

**Fig. 1.**
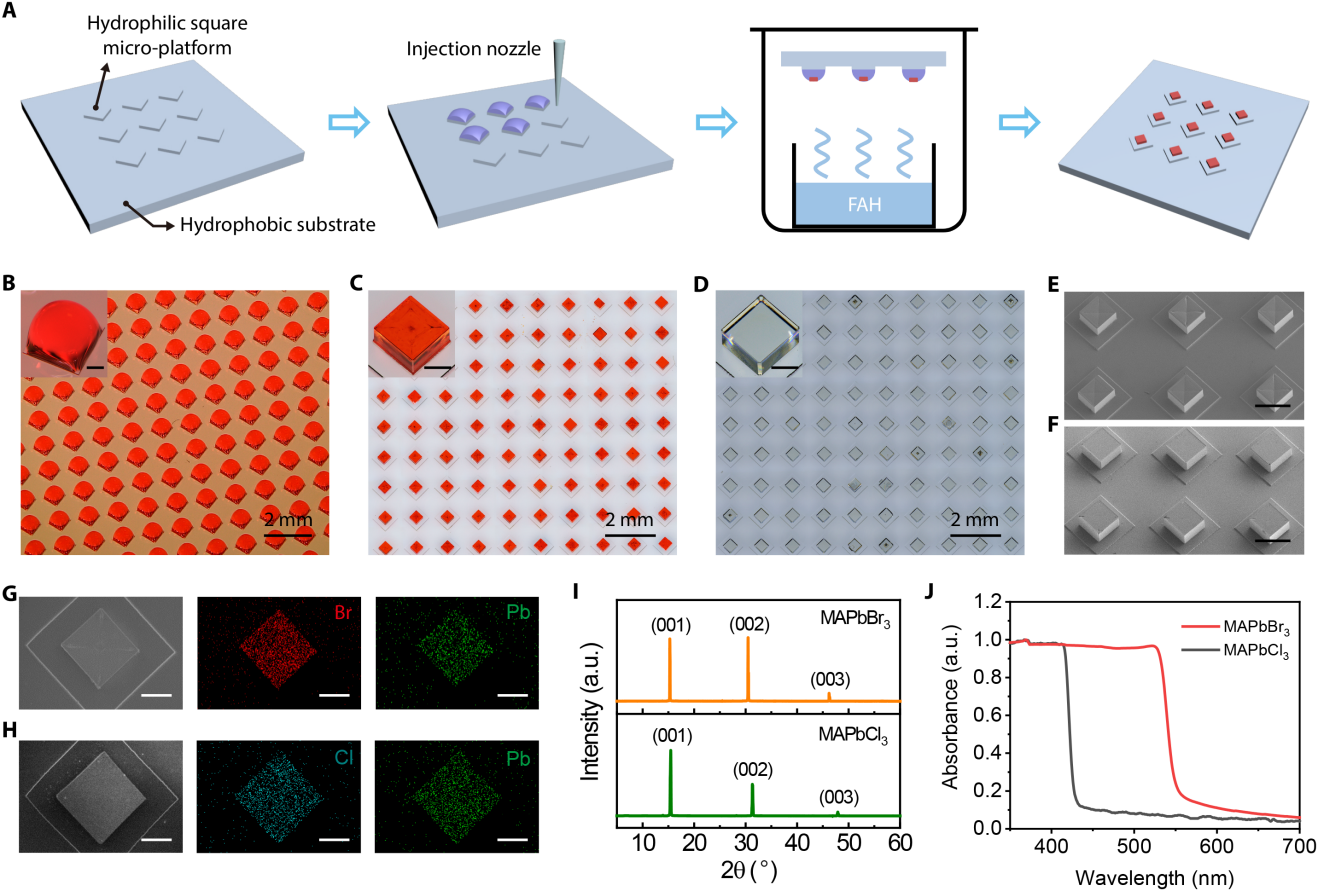
Fabrication and characterization of PSCAs. (**A**) Schematic diagram of the preparation process of PSCAs. Formic acid (FAH) vapor–assisted crystallization was applied. (**B** to **D**) Stereomicroscope images of the dyed red square precursor droplets (B), as-grown methylammonium lead bromide (MAPbBr_3_) crystals (C) and methylammonium lead chloride (MAPbCl_3_) crystals (D). Insets show the detail of the square precursor droplet, MAPbBr_3_ crystal, and MAPbCl_3_ crystal. The scale bars are 200 μm. (**E** and **F**) Scanning electron microscopy (SEM) images of the MAPbBr_3_ crystals (E) and MAPbCl_3_ crystals (F) (tilted view). The scale bars are 500 μm. (**G** and **H**) Energy-dispersive spectroscopy (EDS) element mapping for the MAPbBr_3_ microplate (G) and MAPbCl_3_ microplate (H). The scale bars are 200 μm. (**I**) X-ray diffraction (XRD) pattern of the MAPbBr_3_ arrays and MAPbCl_3_ arrays. (**J**) Absorption spectra of the MAPbBr_3_ arrays and MAPbCl_3_ arrays. a.u., arbitrary units.

The fabrication process of PSCAs is shown in Fig. 1A. Micropillar arrays were formed on the glass substrate by glass etching, and the detailed process is shown in the experimental section. The micropillars were designed as square prisms to limit the shape of the droplets to be square at the bottom. The square prisms were arranged quadrangularly in the form of vertices to vertices, and their width and height are 700 and 20 μm, respectively. Except for the upper surface of the micropillars, all other areas were modified with octadecyltrichlorosilane (OTS) to enhance the hydrophobicity. The contact angles of precursor solution on substrate before and after OTS modification are shown in fig. S1. Precursor solution (160 nl) was precisely dropped onto the center of each micropillar through a homemade dispenser to form precursor droplet arrays, as shown in Fig. 1B. The precursor solution was dyed red for easy observation. The inset image shows that the three-phase contact line is confined by the prism edges, thus limiting the shape of the droplet to be square.

Anti-solvent vapor-assisted crystallization was used by placing precursor droplet arrays and the anti-solvent within a sealed container. Notably, the droplet arrays were positioned upside down during the crystallization process. Three anti-solvents were chosen: dichloromethane (DCM), isopropanol (IPA), and formic acid (FAH). When DCM and IPA were used as anti-solvents, the crystallization outcomes were comparable. In the case of IPA as the anti-solvent, the crystallization results can be observed in fig. S1. For a molar ratio of methylamine bromide (MABr) to lead bromide (PbBr_2_) of 1.0 in *N*,*N*-dimethylformamide (DMF) droplets, the obtained crystals displayed a yellowish color and irregular shapes (fig. S2A). This was due to the higher solubility of MABr in DMF compared to PbBr_2_ (*6*). The consumption of DMF by IPA within the droplet led to faster deposition of PbBr_2_ compared to MABr, resulting in nonstoichiometric MAPbBr_3_. In contrast, with a molar ratio of 1.5, the resulting crystals exhibited a uniform luster and cube-like shapes, as shown in fig. S2B. After complete solvent evaporation, excess MABr precipitated and surrounded the edges of MAPbBr_3_, as shown in fig. S2C. When FAH was used as the anti-solvent, it had unique effects on crystallization. FAH, acting as an atypical anti-solvent, influenced crystallization by altering solvent strength and colloid dissolution (*50*). The evaporation rate of FAH and ambient temperature played crucial roles in crystal quality. The fast FAH evaporation led to rapid crystal growth within suspended droplets, resulting in irregular shapes (fig. S3, A and B). Similar crystallization results were observed at high temperatures (50°C) (fig. S3C). At room temperature, with a carefully controlled FAH evaporation rate, crystals grew at an ideal pace, displaying consistent shapes and superior quality, as evidenced in fig. S3D. In this scenario, most droplets contained only one crystal. This was a result of the limited space within the droplet, where the precipitation of a crystal nucleus rapidly consumed the ions surrounding it, hindering the formation of additional crystal nuclei. Consequently, during crystal growth, the crystals naturally aligned themselves due to the combined influence of gravity of themselves and the surface tension of the droplet, a topic that will be examined in greater detail later. It took ~2 hours for all the droplets to crystallize. Subsequently, the lid of the sealed container was opened and maintained in a partially sealed state to expedite the evaporation of the solvent. Once all solvents had completely evaporated, the PSCAs were successfully obtained on the substrate, which took about 24 hours.

To demonstrate the universality of the strategy, MAPbBr_3_ and methylammonium lead chloride (MAPbCl_3_) arrays were both prepared by this method, as shown in Fig. 1 (C and D). It is obvious that almost all the crystals locate at the exact center of the square prisms, and the four edges of the crystals are parallel to those of the prisms, indicating a high degree of uniformity. Insets show the morphology of MAPbBr_3_ and MAPbCl_3_ microplates viewed at high magnification from a slanted perspective. Scanning electron microscopy (SEM) was performed to investigate the detailed morphology of as-grown PSCAs. The images of the tilted view are shown in Fig. 1 (E and F), and the top views are shown in fig. S4. Both MAPbBr_3_ and MAPbCl_3_ microplates are cubic in shape with smooth side surfaces. The difference is that the upper surface of MAPbCl_3_ is smooth, whereas the upper surface of MAPbBr_3_ has a regular diagonal texture, which is the same as the bulk single crystal (*51*). The fluorescence microscope images provided in fig. S5A reveal distinct regional variations in the fluorescence distribution of MAPbBr_3_. Obviously, the fluorescence intensity at the four edges is visibly higher than that at the center. Conversely, the fluorescence distribution of MAPbCl_3_ (fig. S5B) appears relatively uniform. Energy-dispersive spectroscopy (EDS) was performed to detect the elemental distribution in the MAPbBr_3_ and MAPbCl_3_ microplates. As shown in Fig. 1 (G and H), bromine and lead atoms are homogeneously distributed within the MAPbBr_3_, and chlorine and lead atoms are homogeneously distributed within the MAPbCl_3_. To confirm the crystal structure, x-ray diffraction (XRD) was performed (Fig. 1I). The measurement results reveal the cubic lattice of as-grown MAPbBr_3_ and MAPbCl_3_ microplates, and the peak locations are also consistent with that reported in the literature (*52*). The absorbance spectrum was measured in Fig. 1J and obvious band edge cutoff at 525 nm for MAPbBr_3_ and 414 nm for MAPbCl_3_.

### Crystal array uniformity and self-alignment mechanism

**Fig. 2.**
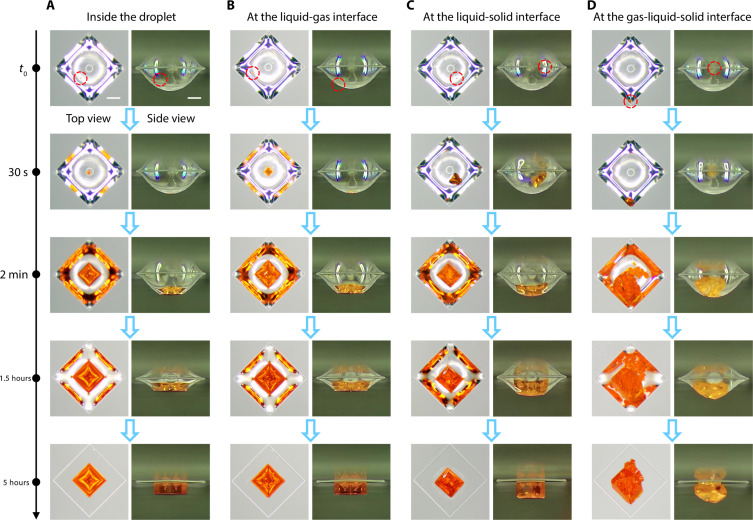
The recording of MAPbBr_3_ microplate growth process. Two cameras synchronously recorded the entire process of crystal growth from the top and side, respectively, and the crystal nuclei may be generated inside the droplet (**A**), at the liquid-gas interface (**B**), at the liquid-solid interface (**C**), and at the gas-liquid-solid interface (**D**). All scale bars are 200 μm.

To explore the self-alignment mechanism of the PSCAs, the crystal growth process was recorded, as shown in Fig. 2. Two cameras synchronously recorded the entire process of MAPbBr_3_ crystal growth from the top and side, respectively. First, among all the observed crystals, crystal nuclei may be generated at four positions: inside the droplet, liquid-gas interface, liquid-solid interface, and gas-liquid-solid interface. The red dashed circles show the location of the crystal nuclei. When the crystal nuclei were generated inside the droplet or at the liquid-gas interface, they would gradually move to the bottom of the suspended droplets under the gravity effect, as shown in Fig. 2 (A and B). When the crystals grew to a certain size, they self-aligned until the four sides of the crystals were parallel to the boundaries of the suspended droplets, which stems from the droplet surface tension. Last, as the solvent evaporated completely, the crystals fell onto the center of the square prisms. The SEM image of the interface between the micropillar and the crystal is shown in fig. S6. Movie S1 shows the entire growth process of the crystal once the nucleus is generated inside the droplet. When the crystal nuclei were generated at the liquid-solid interface or gas-liquid-solid interface, they would be directly attached to the substrate, as shown in Fig. 2 (C and D). The crystals are subject to both gravity and the interaction force between crystals and the solid substrates, which move in opposite directions. As they grow later, there is a chance that they will continue to grow in situ or sink to the bottom of the suspended droplets, which is observed to be a random, not a definite event. In these two cases, there is limited room for crystals to grow, the crystals are hard to grow into cubes, and the final shape and position of the crystals cannot be controlled. Upon the examination of 500 droplets, it was observed that the likelihood of single crystal precipitation and the precipitation of crystal nuclei inside the droplet or at the liquid-gas interface exceeded 95%. Movie S2 recorded the crystal precipitation result of the droplet array with 10 rows and 10 columns. The result shows that the crystal nuclei in 96 droplets were generated either inside the droplet or at the liquid-gas interface and that only four crystals were dead pixels.

To minimize the surface area and reduce the surface energy, the droplet, with its three-phase contact line confined by the prism edges, would spontaneously assume a shape transitioning gradually from square to circular, as depicted in fig. S7A. The droplet surface featured four edges that extend diagonally from the vertex and gradually became less visible. The crystal precipitated in the droplet moved toward the bottom of the droplet under the action of gravity. As the crystal reached the surface of the droplet, its presence altered both the curvature and distribution of edges on its surface, as shown in figs. S7B and S8. Below the crystal, a nearly flat thin liquid surface was created. Conversely, at all four sides of the crystal, there was a distinct increase in curvature on the liquid surface. In addition, the edges on the surface of droplet were no longer aligned along diagonals but instead originated from its vertex and terminated at the vertex of crystal. To reduce the surface energy, the surface tension force (*F*_σ_) of the droplet would drive the crystal to rotate until its four sides align with the edges of the droplet, at which point the droplet has the smallest surface area. *F*_σ_ acted on the four sides of the crystal in contact with the droplet surface and aligned with the direction of the tangent line on the curved liquid surface (fig. S7B). The vertical component of the surface tension force and buoyancy together balanced gravity of the crystal, preventing it from falling out of the droplet. The contact angle between the droplet and crystal is represented by θ. In the vertical direction, we have the following equation

ρcgV=σlvlcosθ+ρlgV (1)

where ρ_c_ and ρ_l_ are the density of crystal and droplet, respectively, *g* is the acceleration of gravity, *V* is the volume of the crystal, σ*_lv_* is the surface tension of the droplet, and *l* is the circumference of the four sides of the crystal. In the horizontal direction, the horizontal component of the surface tension (σ*_lv_*sinθ) acted to pull the crystal toward the corner of the droplet (fig. S7C). The resultant force would propel the crystal clockwise until its four sides align with the edges of the droplet, resulting in a net force of zero.

**Fig. 3.**
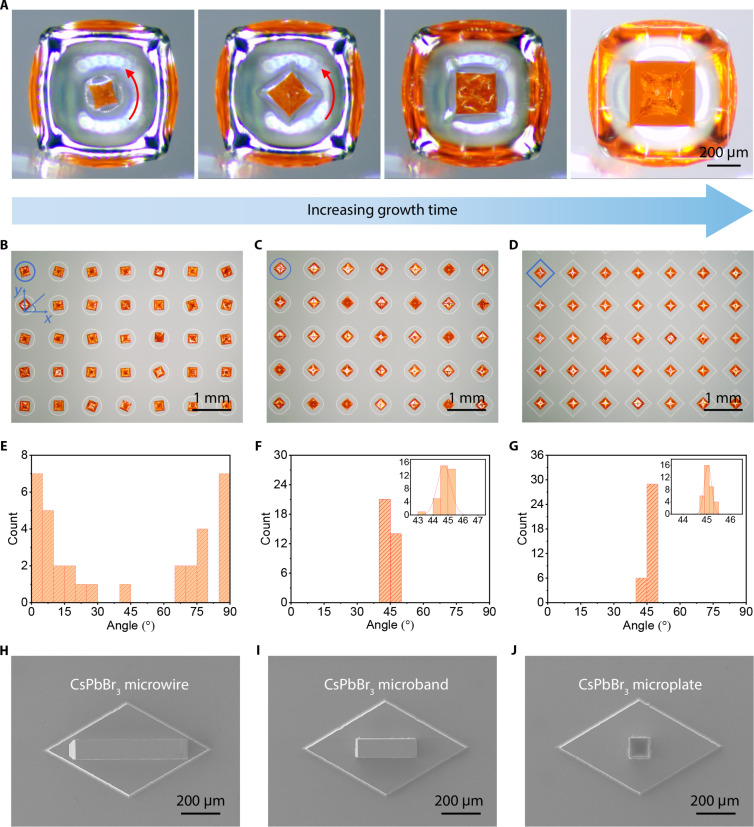
Morphology, position, and directional arrangement control of as-grown PSCAs. (**A**) The recording of MAPbBr_3_ microplate rotation process. (**B** to **D**) Microscopic images of the as-grown MAPbBr_3_ arrays on the rounded square prisms with different corner radiuses. All the width of prisms is 500 μm, and the corner radius is 250 μm for (B) (i.e., circle), 200 μm for (C), 0 μm for (D). (**E** to **G**) The normally distributed results of the angle between the crystals and the *x* axis in (B) to (D). (**H** to **J**) SEM images of as-grown cesium lead bromide (CsPbBr_3_) microwire, microband, and microplate.

The rotation direction of more than 100 crystals was observed, and it was found that the vast majority of them rotated in the direction with the shortest path, that is, the direction of the resultant horizontal component of *F*_σ_. In addition, very occasionally, there will be a case of inverse rotation, as shown in Fig. 3A and movie S3. This is because the capillary flow within the droplet can also have a slight impact on the crystal’s behavior (*53*). As the droplet size decreased, this effect was magnified. In particular, when the droplet size was less than 100 μm, the capillary flow frequently directed crystals toward the droplet’s edge, often resulting in the failure of self-alignment. As shown in fig. S9, when the width of the micropillars was 200 μm, the obtained crystal size was ~80 μm. At this point, some crystals are positioned at the edges of the micropillars, resulting in a lack of directional alignment. Therefore, the droplet-assisted self-alignment strategy described here is particularly well suited for the preparation of PSCAs containing crystals ranging in size from the micron to submillimeter range.

The self-alignment behavior of the crystal was greatly influenced by the shape of the suspended droplet. The droplet shape was restricted by changing the corner radius of the rounded square prism, as shown in fig. S10 (A to C). The width of the rounded square prism was fixed at 500 μm, and the corner radius was 250, 200, and 0 μm, respectively. When the corner radius was 250 μm, the rounded square prism became a cylinder. The MAPbBr_3_ crystallization results are shown in Fig. 3 (B to D). In addition, Fig. 3 (E to G) shows the normally distributed results of the angle between the crystals and the *x* axis in Fig. 3 (B to D). Obviously, the arrangement of the crystals obtained in the circular droplet is disordered (Fig. 3, B and E). This suggests that the circular droplets cannot control the directional arrangement of the crystals. As the droplet corners become sharper, the angle between the crystals and the *x* axis moves closer to 45°. The average angle between the crystals and the *x* axis is 44.7° ± 0.7° in Fig. 3C and 45.2° ± 0.3° in Fig. 3D. This means that the square droplet has greater manipulation impact on the crystal directional arrangement compared to the round droplet. In addition, the average distance between the center of the crystals and the center of the rounded square prisms was also calculated. They are 18.4 ± 10.5 μm for Fig. 3B, 10.1 ± 2 μm for Fig. 3C, and 12.8 ± 4.9 μm for Fig. 3D. For comparison, the average width of the crystals is 242.8 ± 22 μm in Fig. 3D. This suggests that the crystals always end up at the center of the droplets, whether they are round or square. Pentagonal and hexagonal prisms were also developed to constrain the shape of the droplet, as shown in fig. S11 (A and C). The crystallization results of the pentagonal droplets and hexagonal droplets are shown in fig. S11 (B and D). Although the crystals are located at the center of the micropillars, the arrangement of the crystal is disordered. The above experimental results show that the droplet shape plays a crucial role in controlling the directional arrangement of the crystal. Square droplet has the strongest ability to control the directional arrangement of the MAPbBr_3_ crystal, while other shapes, including circular, pentagonal, and hexagonal droplets, have almost no control ability.

The droplet-assisted self-alignment strategy can be applied to crystals of various shapes. In a recent study, Gu *et al.* (*38*) demonstrated the selective printing of perovskite microwire, microband, and microplate arrays by modulating the aggregation of perovskite precursor ions in microdroplets. Similar findings were replicated in our research. By carefully controlling the crystallization temperature and the shape of the droplets, we were able to achieve directional arrangement of cesium lead bromide (CsPbBr_3_) microwires, microbands, and microplates, as illustrated in Fig. 3 (H to J). The precursor droplets were intentionally shaped in a rhombic form, as depicted in fig. S12A, and crystals were allowed to precipitate at different temperatures. At 20°C, the crystals grew into elongated wires (fig. S12B), while, at 40°C, the crystals predominantly formed microbands with some microplates (fig. S12C). The XRD results, shown in fig. S12D, confirmed the orthorhombic lattice structure of as-grown CsPbBr_3_ crystals. The successful preparation of directionally arranged CsPbBr_3_ crystals not only further validates the wide applicability of this strategy but also highlights its flexible diversity. The implementation of this droplet-assisted self-alignment approach opens up fresh possibilities for the controlled growth of crystals with desired configurations and properties, thereby enabling a range of potential applications.

**Fig. 4.**
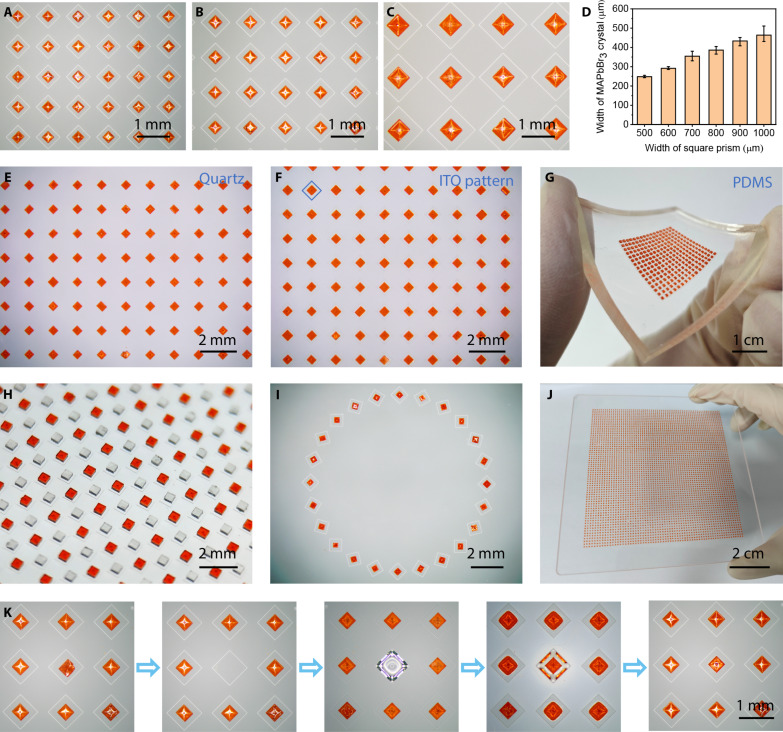
Multi-scene preparation and repair of perovskite crystals. (**A** to **C**) Microscopic images of as-grown MAPbBr_3_ arrays on square prisms with width of 500 μm (A), 600 μm (B), and 1000 μm (C), respectively. (**D**) The dependence of crystal width on the square prism size. (**E** and **F**) Microscopic images of MAPbBr_3_ microplates grown on quartz (E) and ITO pattern (F). (**G**) Optical image of MAPbBr_3_ microplates grown on flexible PDMS substrate. (**H**) Optical image of a PSCA consisting of MAPbBr_3_ and MAPbCl_3_. (**I**) A ring of MAPbBr_3_ microplates. (**J**) Optical image of a MAPbBr_3_ array grown on a 10 cm–by–10 cm substrate. (**K**) The repair process of crystal with uncontrollable shape and position.

Because the larger the micropillar is, the larger the volume of the droplet it can hold, the size of crystals can be controlled by the width of the square prism. Figure 4 (A to C) shows as-grown MAPbBr_3_ arrays on square prisms with widths of 500, 600, and 1000 μm, and the dependence of crystal size on the square prism size is shown in Fig. 4D and fig. S13A. As the width of the square prism increases from 500 to 1000 μm, the width of the crystal increases from ~248 to ~464 μm, and the thickness increases from ~142 to ~258 μm. When the width of the micropillar is 500, 600, 700, 800, 900, and 1000 μm, the ratio of crystal width to thickness is 1.75, 1.80, 1.84, 1.77, 1.81, and 1.80, respectively. The similarity in these width-to-thickness ratios suggests that the crystal size increases uniformly in all three dimensions. In addition, the width of the crystal can be precisely controlled by slightly adjusting the volume of the droplet. For instance, by fixing the width of the square prism at 700 μm, the dependency of crystal size on the volume of the droplet is demonstrated in fig. S13B. As the volume of the droplet increases, the width of the crystal also increases. When the volume of the droplet reaches the maximum amount of liquid that the micropillar can support, which is 195 nl, the width of the crystal attains its maximum value of ~355 μm.

As previously discussed, the self-alignment behavior exhibited by crystals is facilitated by gravity- and surface tension–driven movements and rotations. Notably, this behavior remains unaffected by the nature of the substrate material, thereby enabling the directional alignment of crystals across a diverse range of substrates. Apart from glass micropillar arrays, PSCAs can also be grown on quartz surfaces through the creation of hydrophilic-hydrophobic patterns, as shown in Fig. 4E. In this instance, the hydrophilic region was designed as a square, constraining the precursor droplet to a square shape. Subsequently, the precipitated crystals underwent a similar process, ultimately achieving directional alignment. Furthermore, by etching the ITO layer into a square pattern and applying hydrophobic treatment to the surrounding area, PSCAs can be grown directly on the ITO pattern, providing a solid foundation for the fabrication of integrated devices, as depicted in Fig. 4F. Rigid substrates are not the only option; flexible PDMS can also serve as a promising substrate for wearable electronics. By inverting the mold, micropillar arrays were formed on the PDMS surface, and the precursor solution was dispensed onto each micropillar. The resulting MAPbBr_3_ microplates grown on PDMS are shown in Fig. 4G. The introduction of PDMS offers exciting possibilities for the development of flexible and wearable devices.

Given that the precursor droplets are independent of each other, PSCAs containing MAPbBr_3_ and MAPbCl_3_ can be prepared by controlling the placement of the precursor droplets, as shown in Fig. 4H. In addition, by adjusting the orientation of the droplets, the alignment of the crystals can be tailored accordingly, as demonstrated in Fig. 4I. Large-area preparation is a prominent advantage of arrays, and, as illustrated in Fig. 4J, the area of the PSCAs produced using this method can reach a substantial size of up to 10 cm by 10 cm. This large-area preparation capability holds promise for various applications where scalability and uniformity are paramount. Overall, the versatility and scalability of this method highlight its potential for widespread adoption in materials science and related fields.

### Crystals repair through recrystallization

On the basis of the previous description, the droplet-assisted self-alignment strategy would not be effective when crystal nuclei were formed at the liquid-solid interface or gas-liquid-solid interface. Crystal defects with uncontrollable shapes and positions can be repaired through recrystallization, and the repair process is shown in Fig. 4K. The centrally located crystal has an uncontrollable shape and position (stage 1). First, this crystal was carefully removed under the microscope (stage 2). Then, perovskite precursor solution was dropped onto this micropillar using the homemade dispenser (stage 3). FAH vapor was used to promote crystallization (stage 4). After crystallization, the repaired single-crystal array was obtained (stage 5). To be clear, the substrate in stages 3 and 4 was placed upside down. Figure S14A shows the microscopic image of as-grown MAPbBr_3_ array with 10 rows and 10 columns. The red dashed circles show two crystals with uncontrollable shape and position. After repair, perfect single-crystal array was obtained, as shown in fig. S14B.

### Photodetectors based on MAPbBr_3_ arrays

**Fig. 5.**
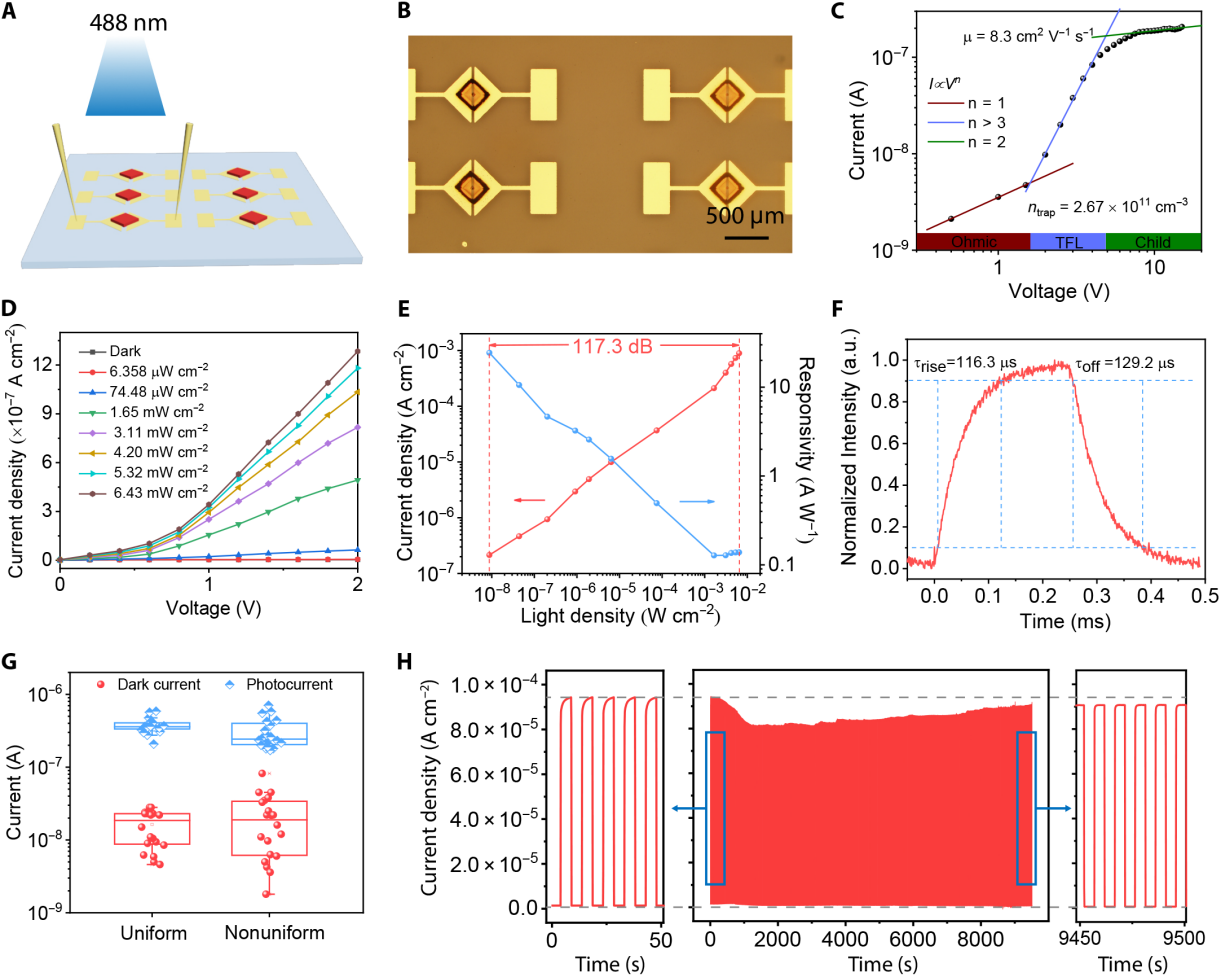
Performances of photodetector based on MAPbBr_3_ arrays. (**A**) Schematic illustration of the photodetector. (**B**) Microscopic image of the MAPbBr_3_ crystals with Au electrodes. (**C**) Current-voltage curve of the photodetector in the dark. TFL, trap-filled-limited. (**D**) Current-voltage curves under different incident light intensities. (**E**) Dependence of the photocurrent density and the responsivity of the device on the light intensity. (**F**) The rise time and decay time of the photodetector. (**G**) Photocurrent and dark current statistics of uniform PSCA and nonuniform PSCA under the bias voltage of 3 V. (**H**) Current density-time curves of a single pixel under the light intensity of 0.11 mW cm^−2^ and bias voltage of 3 V.

To explore the photoelectric properties of the as-grown crystals, photodetectors based on MAPbBr_3_ arrays were fabricated. As shown in the scheme (Fig. 5A), MAPbBr_3_ arrays were grown on the Au electrode pairs, and two probes were used to apply a voltage bias on the electrode pairs. A 488-nm LED was used as a light source to evaluate the photodetection performance. Two right triangle patterns of Au were ingeniously used to restrict the precursor droplet into a square shape. The precipitated crystals underwent movement and rotation for self-alignment. After the solvent was completely volatilized, the crystal got in contact with two electrodes to form a pathway, as shown in Fig. 5B.

First, the space charge–limited current method was used to measure the carrier mobility (μ) and trap density (*n*_trap_) of the MAPbBr_3_ microplate, as depicted in Fig. 5C. The resulting current-voltage (*I-V*) curve exhibited three distinct regions: the ohmic region, the trap-filled region, and the trap-free Child’s region (*54*). The carrier mobility was estimated to be 8.3 cm^2^ V^−1^ s^−1^ by fitting the quadratic Child’s region, while the trap density was deduced to be 2.67 × 10^11^ cm^−3^ from the trap-filled limit voltage. Figure 5D shows the current-voltage curves under different incident light intensities. The current density increases monotonously as increased light intensity, because more photogenerated charge carriers were excited and collected under higher flux intensity. The dependence of the photocurrent density and the responsivity of the device on the light intensity is shown in Fig. 5E. The current density increases from 2.14 × 10^−7^ to 8.90 × 10^−4^ A cm^−2^ as increasing the light intensity from 8.79 × 10^−9^ to 6.43 × 10^−3^ W cm^−2^, and the linear dependence of current density on the light intensity is shown in the figure. The responsivity (*R*) of the photodetector was also calculated with the following formula (*4*)

$$R=\frac{I_{p}-I_{d}}{P}$$ (2)

where *I*_p_ and *I*_d_ are the photocurrent and dark current, respectively, and *P* is the power of the incident light. The responsivity was as high as 24 A W^−1^ at low light intensity of 8.79 × 10^−9^ W cm^−2^. According to the following formula

$$\text{LDR}=20\text{log}\left(\frac{P_{\text{max}}}{P_{\text{min}}}\right)$$ (3)

where *P*_max_ and *P*_min_ are the upper and lower limits of the light intensity, respectively, and the linear dynamic range (LDR) was calculated to be 117.3 dB. The photodetector has a fast response speed with 116.3 μs for rise and 129.2 μs for decay, as shown in Fig. 5F.

To investigate the impact of directional alignment of crystals on device uniformity, we conducted measurements of the photocurrent and dark current distribution for both uniform and nonuniform PSCAs at a bias voltage of 3 V (Fig. 5G). In the case of nonuniform PSCA, the crystals were oriented in various directions (fig. S15), leading to disparities in the effective electrode areas. Consequently, this heterogeneity resulted in a more notable deviation in the current distribution. Conversely, the uniform PSCA exhibited a more consistent current distribution, attributed to the uniform alignment of its constituent crystals. The average dark current of uniform PSCA was 1.64 × 10^−8^ A, while the average photocurrent under a light intensity of 3.46 mW cm^−2^ was 3.74 × 10^−7^ A. These results demonstrate that the device exhibits satisfactory uniformity. To further examine the dynamic photoresponse characteristics of the device, current density-time curves were recorded for a single pixel under a light intensity of 0.11 mW cm^−2^ (3 V bias), as shown in Fig. 5H. After operating for 9500 s (~1000 cycles), there was only a marginal reduction in photoresponse performance, which can be attributed to ion migration within the crystal lattice. Nevertheless, the overall performance of the device remains satisfactory even after prolonged usage.

The environmental and operational stabilities of the perovskite materials are crucial factors to consider. To test the environmental stability, XRD peaks of as-grown MAPbBr_3_ arrays were monitored over 1-year storage without any encapsulations in the atmosphere (fig. S16A). No changes were observed in any of the XRD peaks, indicating the long-term structural stability of the PSCAs fabricated using the droplet-assisted self-alignment strategy. In addition, the operational stability of the fabricated device was also investigated. As shown in fig. S16B, after 1 year in the glove box, the device experienced only a slight photocurrent density decay of ~14%, ensuring its dynamic photoresponse ability even after prolonged exposure to operational conditions. These results indicate that the droplet-assisted self-alignment strategy offers a promising route toward high-quality PSCAs with excellent uniformity, good stability, and satisfactory long-term performance.

The crystal repair process was also found to have an obvious impact on the optoelectronic performance of the crystal. As shown in fig. S17A, the photocurrent density of a dead pixel was considerably lower than that of its surrounding pixels before repair, a result of its relatively poor crystal quality and inadequate contact with the electrodes. However, after the repair process, the photocurrent density of the dead pixel increased to the average level, without notably affecting the surrounding pixels, as displayed in fig. S17B. This result suggests that the repair process can effectively enhance the optoelectronic performance of dead pixels and improve the overall uniformity of the device.

## DISCUSSION

Here, we demonstrate a low-cost and facile droplet-assisted self-alignment strategy to construct precise PSCAs with controllable morphologies, dimensions, positioning, and directional arrangement. This strategy offers a fresh approach to address the challenge of regular arrangement of crystals arising from the inherent randomness and uncertainty of nucleation. Perovskite crystals of various shapes and compositions, including MAPbBr_3_, MAPbCl_3_, and CsPbBr_3_, were precisely aligned through this strategy. Driven by gravity and the surface tension of the droplet, the crystals can self-align to the center of the droplets in the same direction with a yield rate of over 95%. Dead pixel repair can be performed under ambient conditions without notably affecting the surrounding pixels. Flexible PSCAs and excellent photodetector performance are also demonstrated. The proposed method provides the basis for the deployment of PSCAs with a large area, high quality, high precision, high resolution, and high uniformity, which will promote the application of PSCAs in imaging, display, and other optoelectronic applications.

## MATERIALS AND METHODS

### Materials

Methylamine bromide (MABr; 98.0%) and OTS (85%) were purchased from TCI Company. PbBr_2_ (99.0%) was purchased from Aladdin Reagent Ltd. DMF, dimethylsulfoxide (DMSO), methy-lamine hydrochloride (99%), lead dichloride (99%), and cesium bromide (99.9%) were purchased from Adamas. FAH (98%) was purchased from Greagent. All reagents were used as received without further purification. SG2506WC borosilicate glass was purchased from Changsha Shaoguang Chrome Blank Co. Ltd.

### Fabrication of micro-platform arrays

Micropillar arrays were formed on the glass substrate by glass etching (*55*). Briefly, lithography and development were carried out to transfer the pattern on the mask to the Cr layer. The glass substrate was soaked in chromium etchant for 2 min to wash away the exposed chrome. After washing with deionized water, the glass substrate with chrome and photoresist pattern was etched by wet etching in HF:HNO_3_:NH_4_F (1:0.5:0.5 M) solution for 30 min. Then, the substrate was modified by OTS through chemical vapor deposition to make it hydrophobic. Hydrophilic micro-platform arrays with hydrophobic substrate were obtained after immersing the substrate in ethanol and chromium etchant in turn for 2 min to remove the remaining photoresist and chrome.

### Fabrication of PSCAs

The precursor with a molar ratio of 1:1 was dissolved in the corresponding solvent (DMF for MAPbBr_3_ and DMSO for MAPbCl_3_ and CsPbBr_3_). The droplet arrays were obtained by using a homemade dispenser to drop the precursor solution onto micropillars. Then, the substrate containing precursor droplets was placed upside down in a sealed container, which also contained a vial of anti-solvent. Crystals precipitated as the suspended droplets absorbed the anti-solvent vapor. After all the droplets had crystallized, the lid of the sealed container was opened and maintained in a partially sealed state to expedite the evaporation of the solvent. PSCAs were obtained as the solvent was completely volatilized.

### Fabrication of photodetector devices based on MAPbBr_3_ PSCAs

Cr/Au with the thickness of 3 nm/20 nm were deposited on the quartz substrate. Photolithography, Au etching, and Cr etching were performed successively to obtain the electrode pair arrays. Then, the exposed quartz substrate was hydrophobically modified. A certain amount of perovskite precursor solution was accurately dropped onto the electrode pair. The electrode pair constrained the precursor droplet into a square shape. The precipitated crystals underwent movement and rotation for self-alignment. After the solvent was completely volatilized, the crystal fell on the two electrodes to form a pathway.

### Measurement and characterization

The stereomicroscope images were taken by a DSX1000 digital microscope. The fluorescence microscope images were taken by an OLYMPUS FLUVIEW FV3000 laser scanning confocal microscope. The SEM images and the EDS element mappings were taken by a JEOL FESEM 6700F electron microscope with primary electron energy of 3 kV, and the surfaces were sputter-coated with 2-nm Au before testing. The XRD measurement was performed by Rigaku x-ray diffractometer (SmartLab 3). The ultraviolet-visible absorption spectra were collected by UV-2600 spectrophotometer operating from 400 to 700 nm. Two industrial digital cameras were used to record the crystal growth processes. The current signals under dark and light are collected by Keithley 2400 source meter. The light source used is 488-nm LED, which is corrected by silicon diode to obtain light intensity. The response time is collected by oscilloscope and SR570 low-noise current preamplifier.
