# Supplementary material for: Precise arraying of perovskite single crystals through droplet-assisted self-alignment
Source: Sci Adv. 2024 Jul 10;10(28):eado0873. doi: 10.1126/sciadv.ado0873 (PMC11235166; doi:10.1126/sciadv.ado0873)
Supplement: Supplementary file 1 — Figs. S1 to S17 Legends for movies S1 to S3 [file sciadv.ado0873_sm.pdf]

Science Advances
AAAS

Supplementary Materials for

Precise arraying of perovskite single crystals through droplet-assisted self-alignment

Jianglei Zhang *et al.*

Corresponding author: Junhu Zhang, zjh@jlu.edu.cn; Haotong Wei, hweichem@jlu.edu.cn

Sci. Adv. **10**, eado0873 (2024)
DOI: 10.1126/sciadv.ado0873

The PDF file includes:

Figs. S1 to S17
Legends for movies S1 to S3

Other Supplementary Material for this manuscript includes the following:

Movies S1 to S3

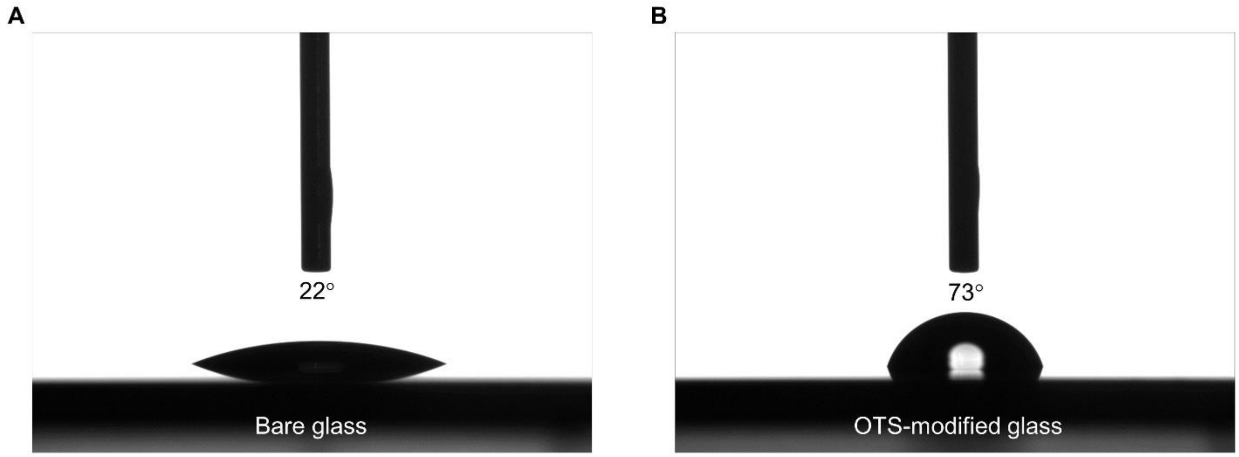

Fig. S1.

Contact angles of $MAPbBr_3$ precursor solution on bare glass (A) and OTS-modified glass (B).

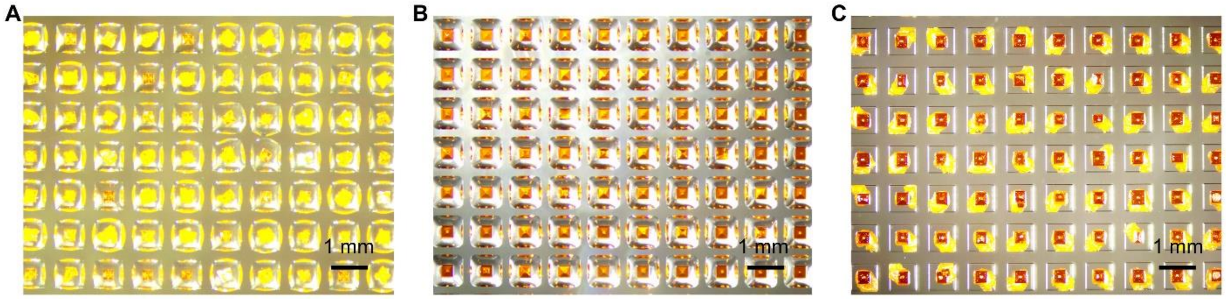

Fig. S2.

Crystallization results when isopropanol was selected as the anti-solvent. (**A** and **B**) Microscopic images of $MAPbBr_3$ within the suspended droplets with the molar ratio of MABr to $PbBr_2$ of 1.0 (A) and 1.5 (B). (**C**) Microscopic image of $MAPbBr_3$ crystals after solvent complete evaporation in (B).

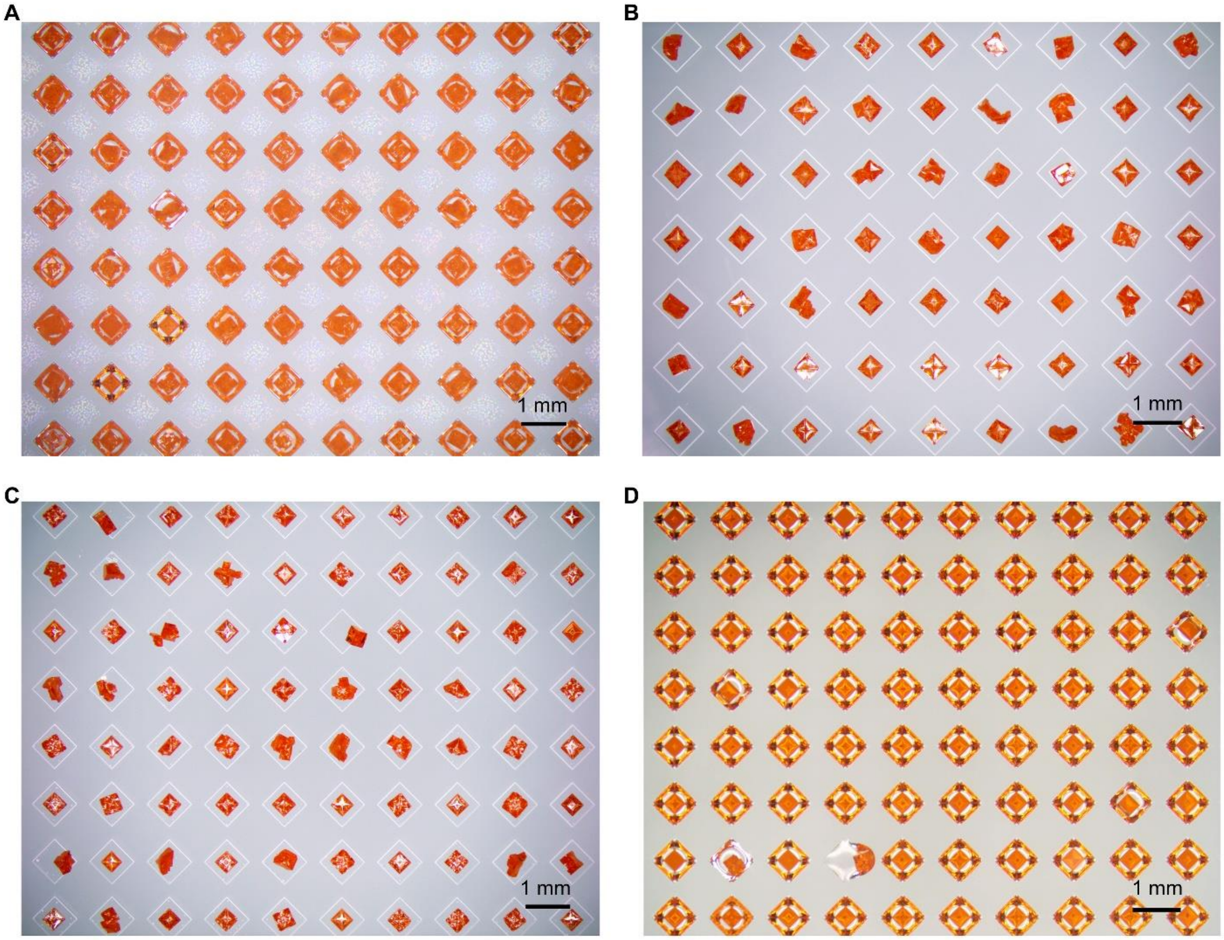

Fig. S3.

Crystallization results with different evaporation rates of FAH and ambient temperatures. (**A** and **B**) Microscopic images of $MAPbBr_3$ within the suspended droplets (A) and $MAPbBr_3$ after solvent complete evaporation (B) with rapid evaporation rate of FAH. (**C**) Microscopic image of $MAPbBr_3$ grown at 50℃. (**D**) Microscopic image of $MAPbBr_3$ within the suspended droplets grown at room temperature with controlled FAH evaporation rate.

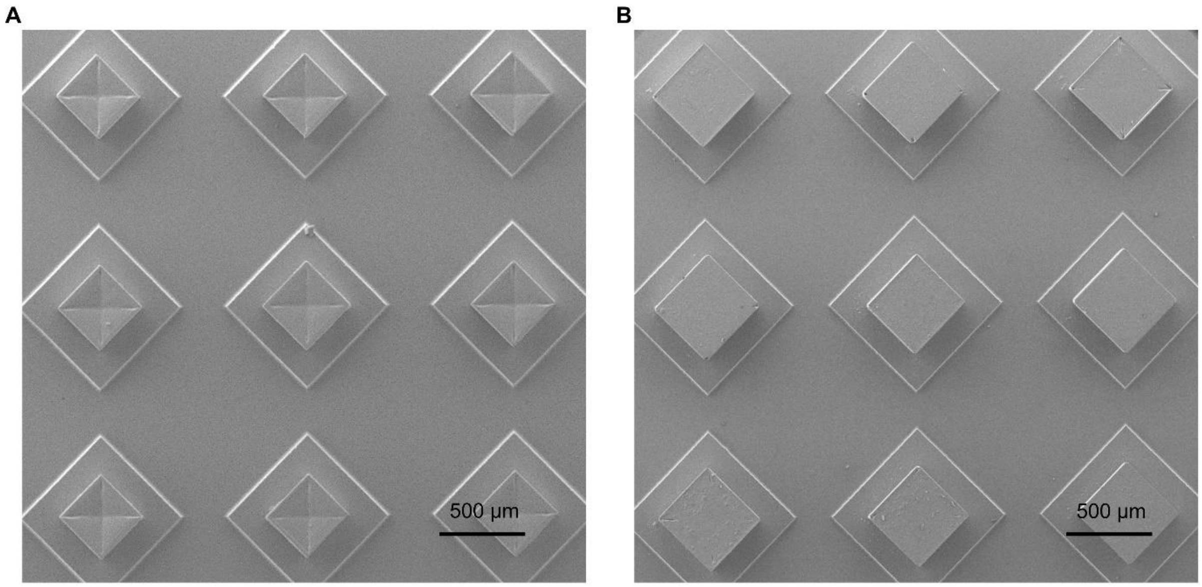

Fig. S4.

SEM images of as-grown $MAPbBr_3$ arrays (A) and $MAPbCl_3$ arrays (B) on the square prisms (top view).

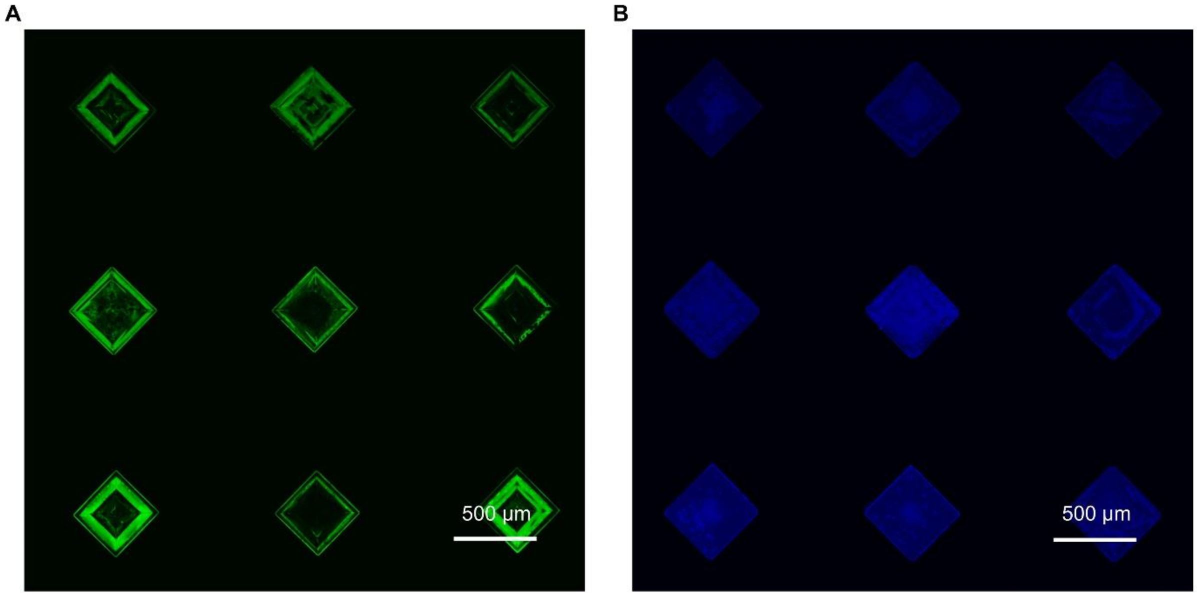

Fig. S5.

Fluorescence microscope images of as-grown $MAPbBr_3$ arrays (A) and $MAPbCl_3$ arrays (B) (which are excited with a pulsed 405 nm laser).

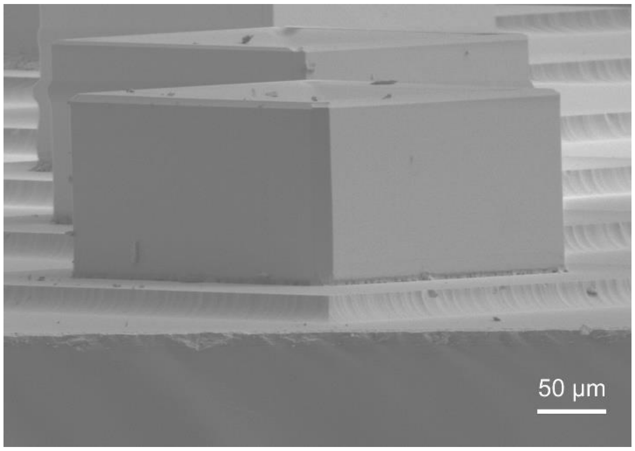

Fig. S6.
SEM image of the $MAPbBr_3$ crystal at the center of the micropillar (side view).

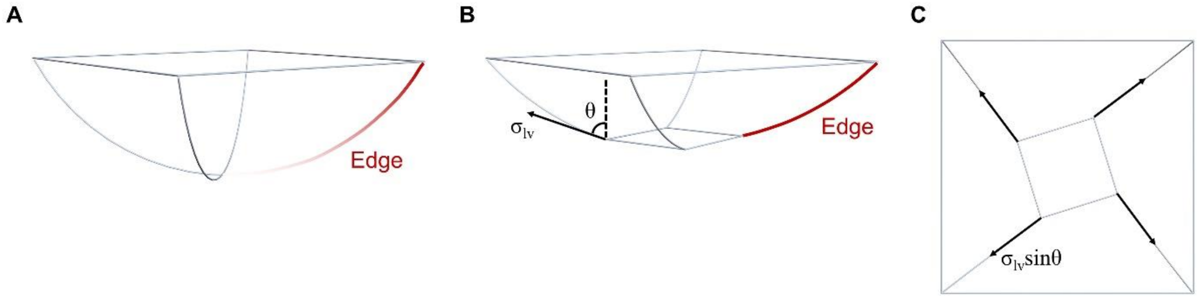

Fig. S7.

Schematic diagram of the crystal self-alignment process. (**A**) Schematic diagram of a square suspended droplet with four edges. (**B**) Schematic diagram of the droplet shape altered by the crystal. (**C**) Schematic diagram of the crystal subjected to the surface tension of the droplet. (top view).

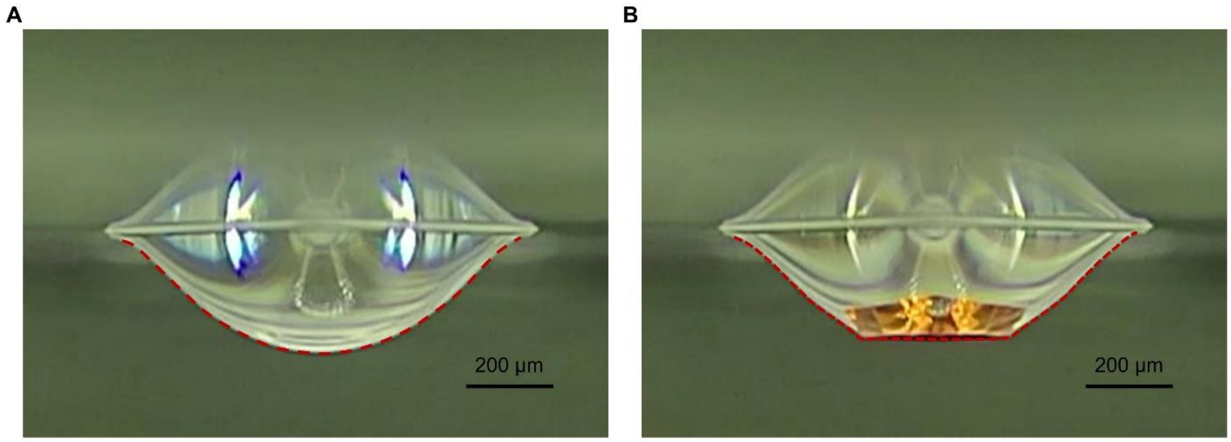

Fig. S8.

The shape of the droplet altered by the crystal. (**A** and **B**) Microscopic images of the suspended droplet without (A) and with (B) a crystal. (side view). The dotted red lines outline the droplets.

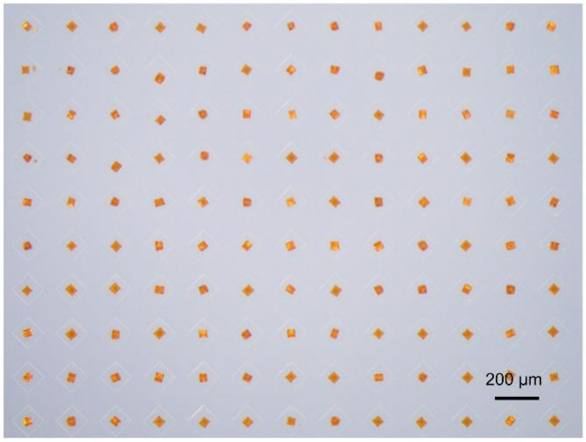

Fig. S9.
Microscopic images of the $MAPbBr_3$ arrays with crystal width about 80 μm.

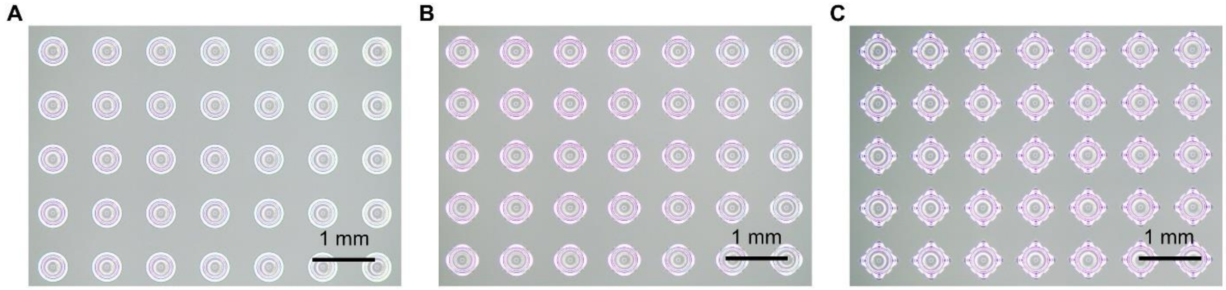

Fig. S10.

Microscopic images of the suspended droplets on the rounded square prisms with different corner radiuses. (**A** to **C**) All the width of prisms is 500 μm and the corner radius is 250 μm for (A) (i.e., circle), 200 μm for (B), 0 μm for (C).

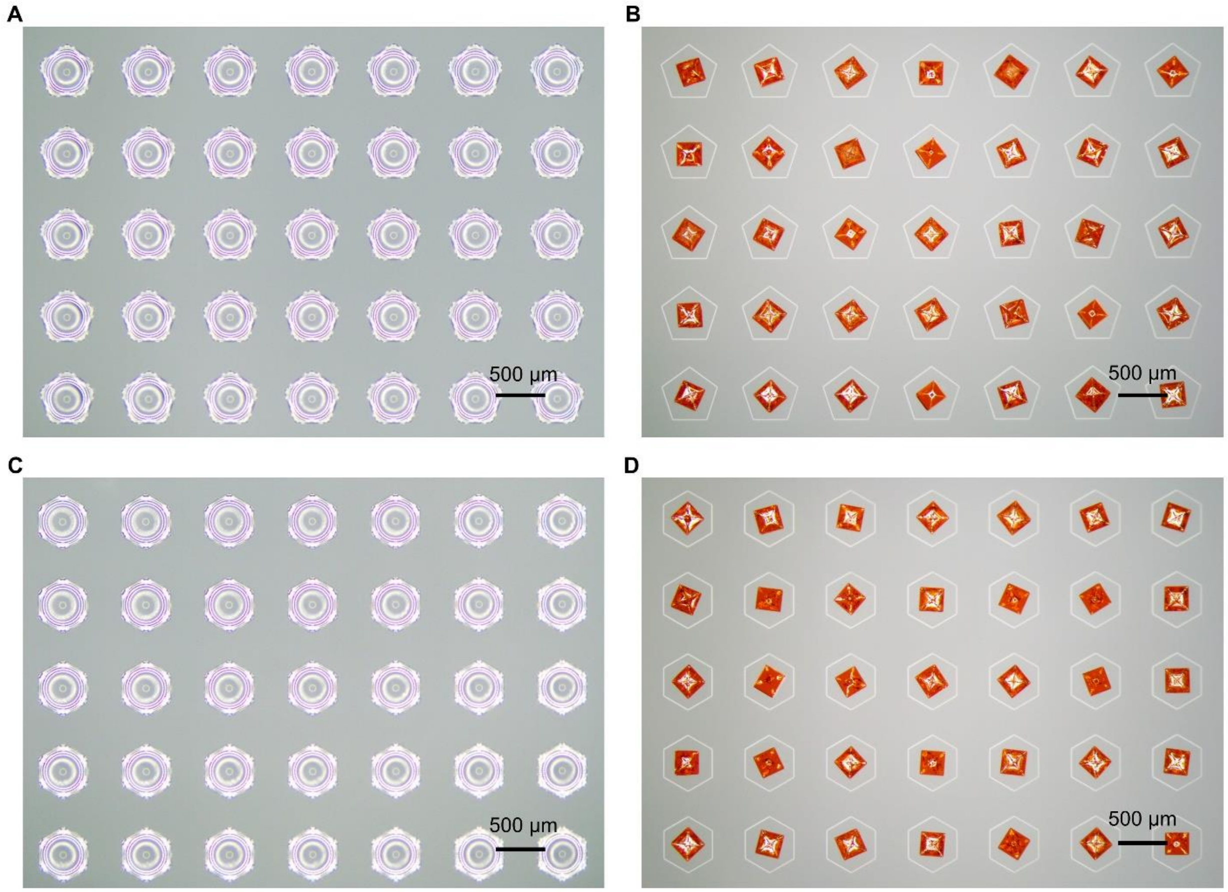

Fig. S11.

Crystal arrangement results on the pentagonal prisms and hexagonal prisms. (**A** and **B**) Microscopic images of the suspended droplets (A) and $MAPbBr_3$ arrays (B) on the pentagonal prisms. (**C** and **D**) Microscopic images of the suspended droplets (C) and $MAPbBr_3$ arrays (D) on the hexagonal prisms.

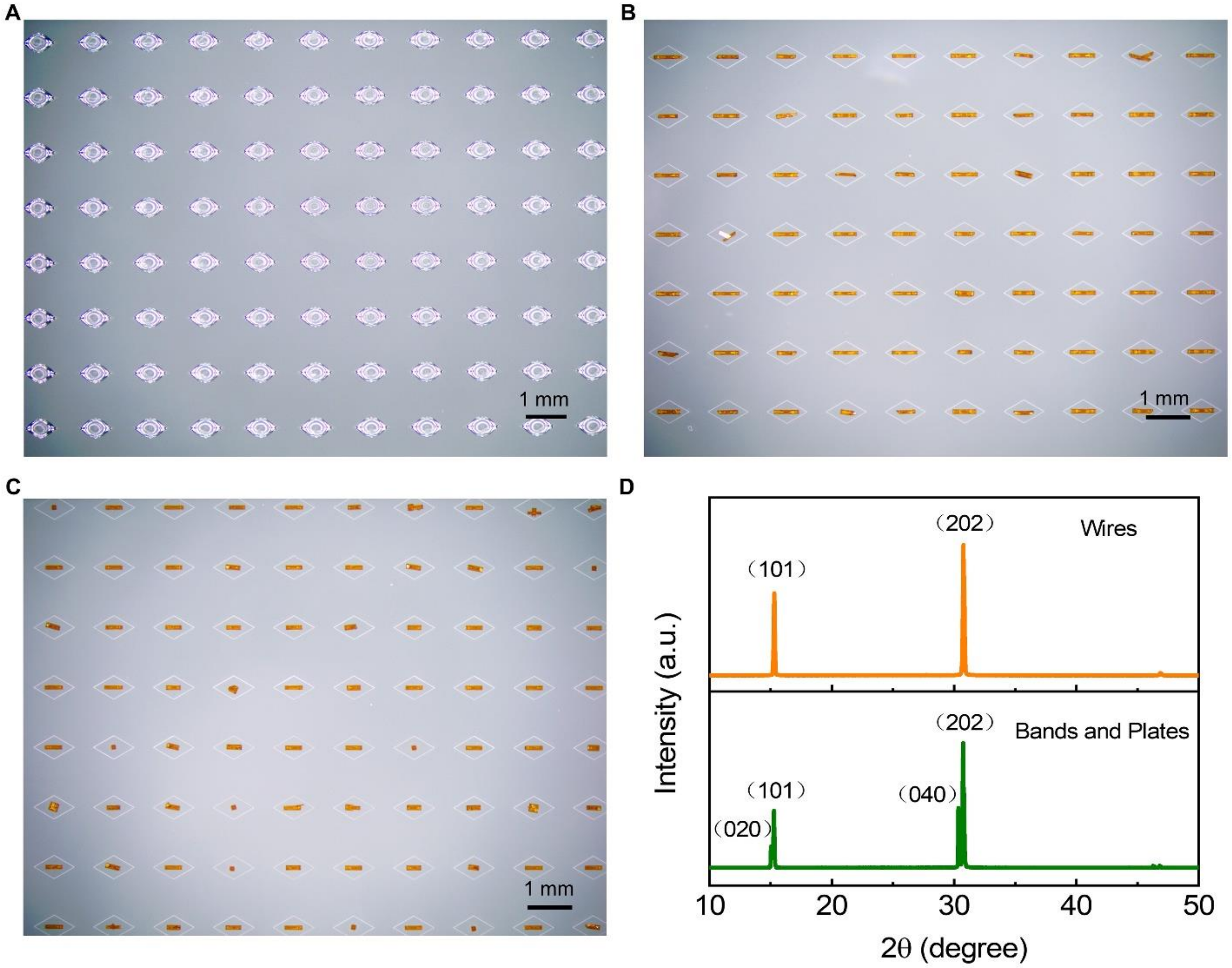

Fig. S12.

Directional arrangement of $CsPbBr_3$ crystals. (**A**) Microscopic image of the rhombic precursor droplets. (**B**) Microscopic image of the $CsPbBr_3$ microwires grown at 20℃. (**C**) Microscopic image of the $CsPbBr_3$ microbands and microplates grown at 40℃. (**D**) XRD pattern of the $CsPbBr_3$ microwires, microbands and microplates.

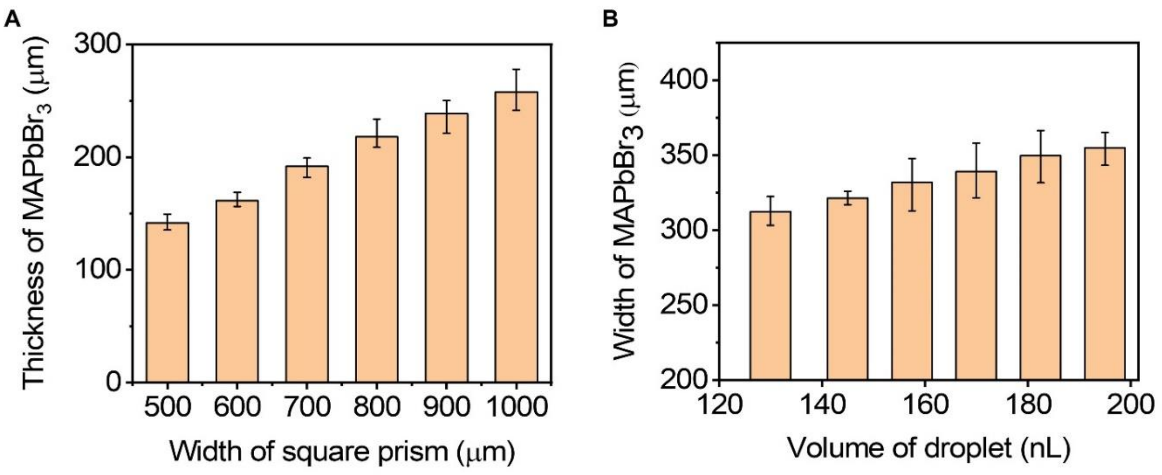

Fig. S13.

The dependence of crystal size on the square prism size. (**A**) The dependence of crystal thickness on the square prism width. (**B**) The dependence of crystal width on the volume of precursor droplet.

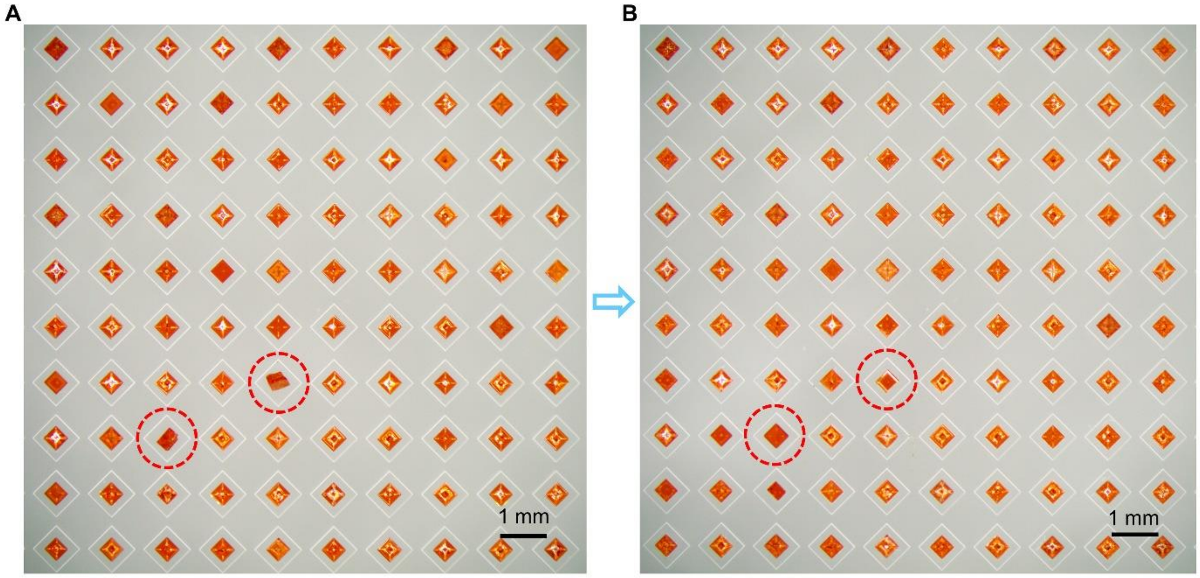

Fig. S14.

Repair of dead pixels. (**A**) Microscopic image of the as-grown $MAPbBr_3$ arrays with 10 rows and 10 columns. The red dotted circles show the crystals with uncontrollable shape and position. (**B**) Microscopic image of the repaired $MAPbBr_3$ arrays.

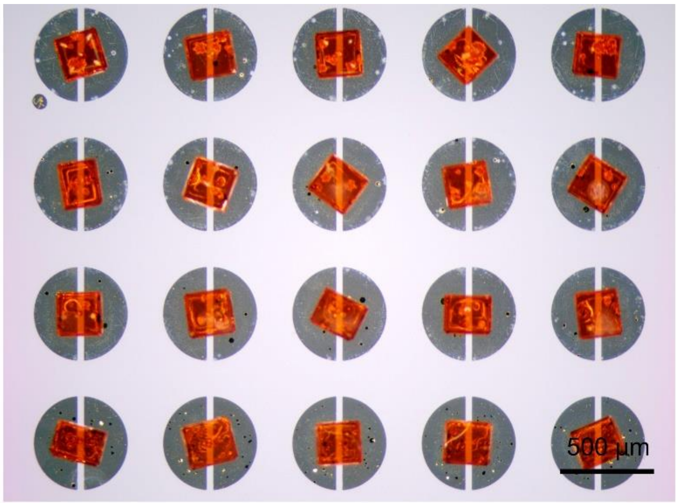

Fig. S15.
Microscopic image of the non-uniform PSCA with Au electrodes.

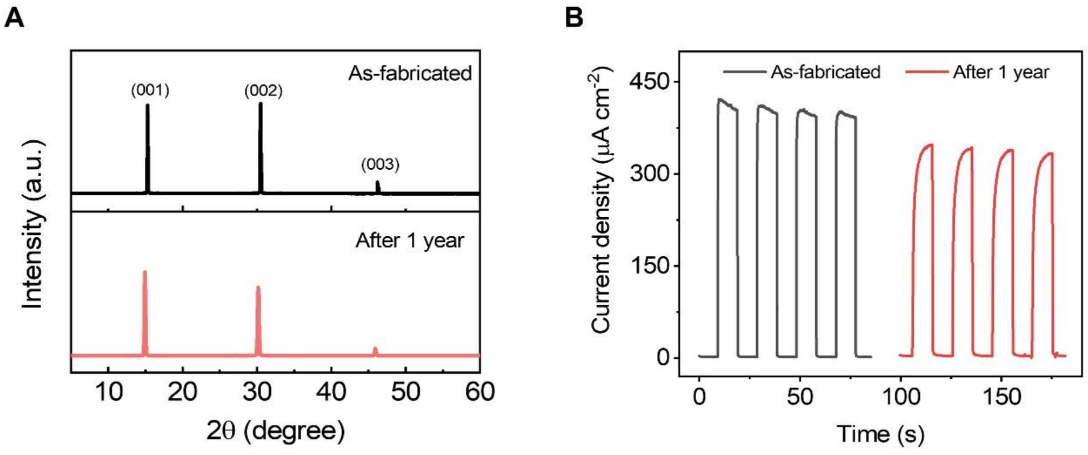

Fig. S16.

Environmental stability of the detectors. (**A**) XRD peaks of the $MAPbBr_3$ arrays within one year in the atmosphere. (**B**) Current density-time curves of the detector within one year in the glovebox (light intensity of 3.1 mW cm^{-2} and bias voltage of 3 V).

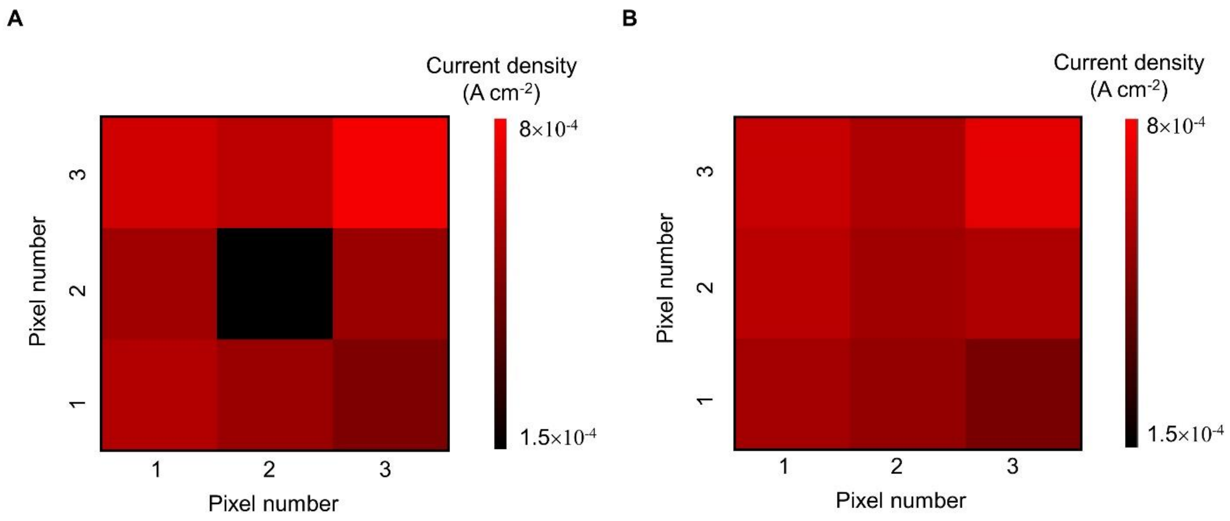

Fig. S17.

Photocurrent density distribution imaging results before (A) and after (B) the repair of the dead pixel. The bias voltage was 3V and the light intensity was 3.46 mW cm^{-2}. After repairing, the photocurrent density of dead pixel increased from 1.5×10^{-4} to 5.52×10^{-4} A cm^{-2} with no obvious difference from the surrounding pixels.

Movie S1.
The entire growth process of the crystal once the nucleus is generated inside the droplet.

Movie S2.
The crystal precipitation results of the droplet array with 10 rows and 10 columns.

Movie S3.
The movement and self-alignment of crystal in the suspended droplet.
